# Four-dimensional computed tomography evaluation of shoulder joint motion in collegiate baseball pitchers

**DOI:** 10.1038/s41598-022-06464-5

**Published:** 2022-02-25

**Authors:** Daisuke Momma, Alejandro A. Espinoza Orías, Tohru Irie, Tomoyo Irie, Eiji Kondo, Norimasa Iwasaki, Nozomu Inoue

**Affiliations:** 1 Center for Sports Medicine, Hokkaido University Hospital, Kita 14, Nishi 5, Sapporo, Hokkaido 060-8638 Japan; 2 Department of Orthopedic Surgery, Rush University Medical Center, Chicago, IL USA; 3 Department of Orthopaedic Surgery, Faculty of Medicine and Graduate School of Medicine, Hokkaido University, Sapporo, Japan

**Keywords:** Bone, Bone imaging

## Abstract

The purpose of this study is to evaluate the glenohumeral contact area, center of glenohumeral contact area, and center of humeral head during simulated pitching motion in collegiate baseball pitchers using four-dimensional computed tomography (4D CT). We obtained 4D CT data from the dominant and non-dominant shoulders of eight collegiate baseball pitchers during the cocking motion. CT image data of each joint were reconstructed using a 3D reconstruction software package. The glenohumeral contact area, center of glenohumeral contact area, center of humeral head, and oblateness of humeral head were calculated from 3D bone models using customized software. The center of glenohumeral contact area translated from anterior to posterior during maximum external rotation to maximum internal rotation (0.58 ± 0.63 mm on the dominant side and 0.99 ± 0.82 mm on the non-dominant side). The center of humeral head translated from posterior to anterior during maximum external rotation to maximum internal rotation (0.76 ± 0.75 mm on the dominant side and 1.21 ± 0.78 mm on the non-dominant side). The increase in anterior translation of the center of glenohumeral contact area was associated with the increase in posterior translation of the center of humeral head. Also, the increase in translation of the center of humeral head and glenohumeral contact area were associated with the increase in oblateness of the humeral head. 4D CT analyses demonstrated that the center of humeral head translated in the opposite direction to that of the center of glenohumeral contact area during external rotation to internal rotation in abduction in the dominant and non-dominant shoulders. The oblateness of the humeral head may cause this diametric translation. 4D CT scanning and the software for bone surface modeling of the glenohumeral joint enabled quantitative assessment of glenohumeral micromotion and be used for kinematic evaluation of throwing athletes.

## Introduction

Glenohumeral joint micromotion and shoulder internal impingement are the important factors of shoulder pathology in throwing athletes^1^. Pitching in baseball has been described as six discrete phases: wind up, stride, cocking, acceleration, deceleration, and follow through^2^. The cocking and acceleration phases are most commonly implicated in shoulder pathology of throwing, due to excessive abduction and external rotation of the glenohumeral joint^3^. Numerous studies have conducted overhead throwing analysis^4–7^, but in vivo glenohumeral joint kinematics during pitching motion remains controversial because it is difficult to directly assess micromotion of the glenohumeral joint^8,9^.

Glenohumeral joint contact patterns well reflect pathological conditions such as rotator cuff tear and glenohumeral joint instability^10,11^. Based on this theory, Bey et al. reported that joint contact patterns are not only a more sensitive measurement than conventional kinematics for detecting subtle differences in joint function but may also provide a more clinically relevant indication of the extent to which a conservative or surgical procedure has adequately restored normal joint function^12^. Therefore, measurement of glenohumeral joint contact patterns can reveal the kinematics of the glenohumeral joint in baseball players.

Measurement of glenohumeral joint mechanics is a challenging task, especially under in vivo conditions. The glenohumeral joint kinematics of normal shoulders have been evaluated using cadaveric specimens^13^, radiographs^14^, fluoroscopy^15^, magnetic resonance imaging^16,17^, and electromagnetic tracking devices^18^. However, there are limitations associated with these conventional measuring techniques. In particular, cadaveric studies cannot accurately simulate in vivo conditions because the muscle forces and joint forces are unknown. Four-dimensional computed tomography (4D CT) enables these issues to be overcome, and can be used for the clinical evaluation in various fields, with a low radiation dose^19^. Previous studies have shown the potential of dynamic assessment with 4D CT technology for assessing complex pathologies and their underlying mechanisms^20,21^. We hypothesized that glenohumeral joint kinematics during simulated pitching motion could be quantitatively analyzed in vivo using a 4D CT device. The purpose of this study was to evaluate the glenohumeral contact area, center of glenohumeral contact area, and center of humeral head during simulated pitching motion in baseball players by 4D CT analysis.

## Methods

### Ethics statement

Our study was carried out in accordance with relevant guidelines of Hokkaido University Hospital and approved by the Research Ethics Review Committee of Hokkaido University Hospital. Our research protocols for human samples used in this study was approved by the Research Ethics Review Committee of Hokkaido University Hospital (approval ID: 011–0327). Informed consents for the use of samples in our research were obtained from all participants and their guardians. Informed consents for publication of identifying images in an online open-access publication were also obtained.

### Subjects

We obtained 4D CT images of the dominant and non-dominant shoulders of eight baseball players as they performed the cocking motion. All participants were competitive level collegiate baseball pitchers. The participants were all male overthrow or three quarter throw pitchers, without a history of shoulder complaints, with normal shoulder examination with no pain on shoulder joint palpation, and negative subacromial impingement test results. The years of baseball experience was determined by mean number of years on the baseball team. Body weight and height were recorded, and the shoulder range of motion was measured using a goniometer. We measured the passive glenohumeral range of motion (ROM) in external rotation (ER) and internal rotation (IR) at 0 degree of shoulder abduction in the supine position with restriction of the scapulothoracic movement in the dominant and nondominant shoulders for first ER and IR. We also measured the passive glenohumeral ROM in ER and IR at 90 degree of shoulder abduction in the supine position with restriction of the scapulothoracic movement in the dominant and nondominant shoulders for second ER and IR.

### 4D CT image data acquisition

**Figure 1:**
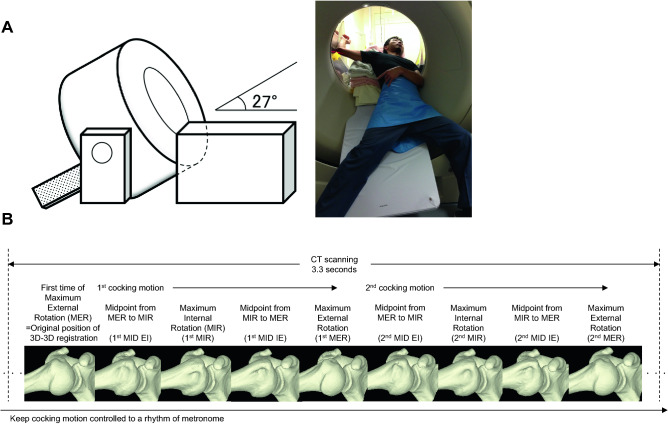
4D CT scanning of the shoulder joint during simulated pitching motion and definition of each shoulder position. (**A**) 4D CT image acquisition, the maximum angle between the board and the ground was 27° and the patient rotates the shoulder in the computed tomography gantry, (**B**) definition of each shoulder position through 3.3 s scanning time of two cocking motions controlled to a rhythm of metronome. MER, maximum external rotation; MIR, maximum internal rotation; MID EI, midpoint from MER to MIR; MID IE, midpoint from MIR to MER.

All images were obtained with a 320-slice multidetector 4D CT scanner (Aquilion One; Toshiba Medical Systems, Tochigi, Japan) with a wide field-of-view (FOV), 0.5-mm slice thickness, and gantry tilt of 22°. The maximum board tilt was 27 degrees to scan shoulder motion. (Fig. 1A). CT images were obtained with shoulder abduction of 90° and elbow flexion of 90°. The scan parameters were set as follows: dynamic volume scan; wide FOV (size, LL; detector width, 16 cm); 80 kV; 100 mA; gantry speed, 0.275 s/rotation; effective mAs, 27; reconstruction function, FC01; reconstruction rate, 0.1 s (31 volume); AIDR, standard; slice thickness, 0.5 mm; and slice interval, 0.5 mm. The scanning time was 3.3 s for two cocking motions controlled to a rhythm of 80 beats/min with a metronome. Each shoulder positions were defined from 3D bone model (Fig. 1B). The total radiation exposure was set not to exceed 2.4 mSv.

### Analysis of dynamic motion of glenohumeral joint

**Figure 2:**
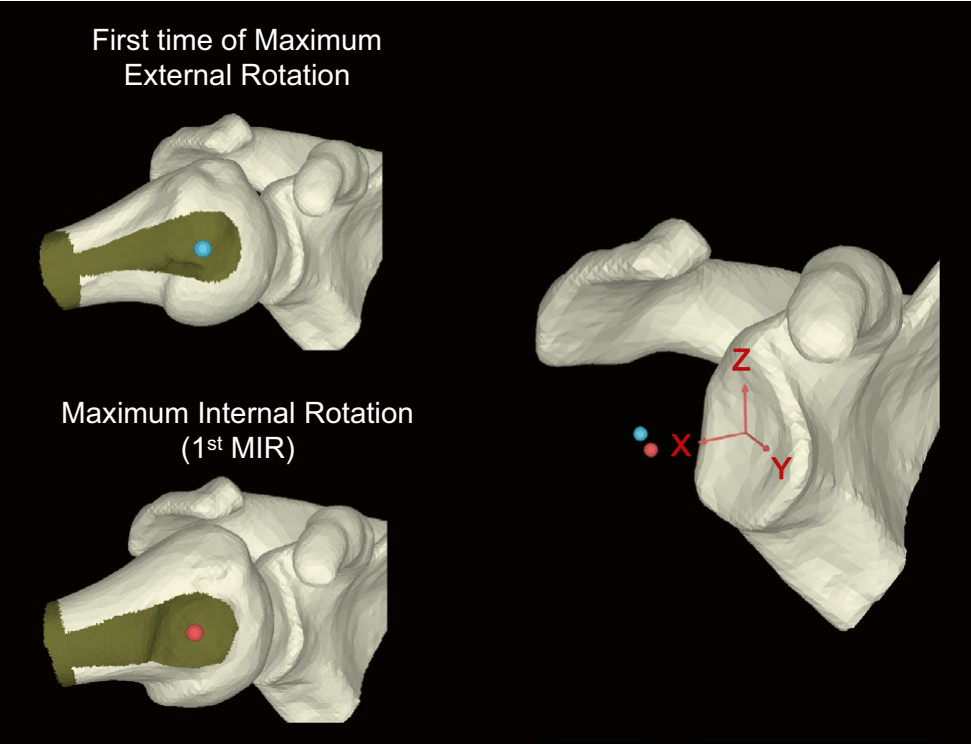
Coordinate system for the direction of translation of the joint contact area and humeral head centroid. Blue sphere; center of humeral head (CHH) at the original position, Red sphere; CHH at the first maximum internal rotation. The axes of the scapula coordinate system are aligned in the lateral/medial (X axis), anterior/posterior (Y axis), and superior/inferior (Z axis) directions.

CT image data of each shoulder joint were imported in DICOM format and segmented using a segmentation software package (Mimics 21R; Materialise, Leuven, Belgium). Images were reconstructed to 3D scapula and humerus bone models, and the resulting 3D models were then exported as pointcloud and polygon models using the same software package. The bone thresholds were determined by referencing the previous studies of the 3D bone model^22^. These 3D scapula and humerus bone models were then analyzed with a customized software created in Microsoft Visual C +  + with Microsoft Foundation Class programming environment (Microsoft, Redmond, WA) for further analysis^23,24^. The glenohumeral contact area, center of glenohumeral contact area, and center of humeral head were analyzed with a customized software^25^. Articular contact areas were defined as areas where the least distances were under a certain threshold level. The distance thresholds were determined by referencing the previous studies of the distance of the shoulder joint space^11^; these thresholds were 4.0 mm in the glenohumeral joint. We normalized using body height and glenoid total area (data not shown). A centroid of the contact area was projected on the glenoid surface and defined as a center of the contact area. The center of humeral head was calculated according to a similar technique described by Yanke et al^22^. Briefly, a midpoint of the humeral head and the anatomical neck centroids was calculated and defined as an initial temporal humeral head center. Distances between the temporal humeral head center and each point of the humeral head pointcloud model were calculated, and a standard deviation of all distances was calculated. The temporal humeral head center was moved within a search range of ± 5.0 mm from the aforementioned humeral head centroid in x, y, and z directions in 1.0-mm increments until the standard deviation of the distance became the smallest. A new search range of ± 0.5 mm from this temporal humeral head center was set, and the procedure was repeated in 0.1-mm increments. The temporal humeral head center with the smallest standard deviation of the distances was defined as the final humeral head center. The major and minor axes of humeral head were also analyzed with a customized software^22^. Oblateness of humeral head was defined as (length of major axis—length of minor axis) / length of major axis. The original position was defined as the first frame of maximum external rotation (MER) (Fig. 1B). A validated 3D-3D registration method (accuracy, translation: 0.1 mm, rotation: 0.2°) was used for the glenoid and the humeral head between the original position and each rotated position to obtain a transformation matrix from the original position to each rotated position to calculate translation of the center of glenohumeral contact area and the center of humeral head at each rotated position^26,27^. We used the local 3D coordinate system of the glenoid using the individual bony landmarks, modified the anatomical coordinate system for the shoulder^28^ (Fig. 2). The glenoid plane was defined as the best-fitting plane of the glenoid surface. The glenohumeral contact area, center of glenohumeral contact area, and center of humeral head were relative to the scapular coordinate system.

### Statistical analysis

An a priori power analysis (G*Power software) indicated that a sample of 8 participants would be appropriate to establish a statistical power of 0.95, at the predetermined a level of 0.05, and with a large effect size of 0.85. We compared the glenohumeral contact area, oblateness of humeral head, translation of center of glenohumeral contact area, and translation of center of humeral head between the dominant and non-dominant sides using paired t test. One-way repeated-measures analysis of variance was used to investigate change in the glenohumeral contact area, translation of center of glenohumeral contact area, and center of humeral head per frame, The correlation between the translation of center of glenohumeral contact area and center of humeral head was examined. The correlation between the translation of center and humeral head oblateness was also examined. *P* values < 0.05 were considered significant. Data are presented as the mean ± SD and corresponding 95% confidence intervals.

## Results

### Participant demographics

4D CT imaging of both shoulders was performed in 8 male college volunteers (age, 18.6 ± 0.5 years; range, 18–19 years). There was no significant difference in the mean first ER or second IR between the dominant and non-dominant sides (Table 1). Mean second ER was significantly greater on the dominant side than on the non-dominant side (p = 0.0179). In our bone model, maximum external rotation averaged 112 degrees, and the mean maximum internal rotation was 78 degrees.

**Table 1:** Participant Characteristics.

Age, y	Dominance	Experience, y	Height, cm	Weight, kg	Range of motion (Dominant/Non-Dominant), deg				Humeral head oblateness	
					First ER	First IR	Second ER	Second IR	Dominant	Non-Dominant
19	R	11	187	82	71/66	T12/T5	117/102	31/39	0.078	0.076
19	R	7	181	72	79/54	T6/T5	113/88	59/81	0.091	0.074
18	L	9	169	64	82/72	T3/T5	114/104	27/32	0.091	0.090
18	L	8	172	70	43/43	T8/T5	96/86	28/38	0.122	0.110
18	R	11	180	70	81/81	T5/T3	101/111	62/58	0.113	0.067
19	L	7	176	63	91/91	T12/T5	99/94	33/52	0.062	0.087
19	R	10	172	70	48/48	T10/T6	103/103	31/39	0.090	0.072
19	R	12	180	70	55/50	T5/T3	107/87	49/56	0.087	0.077
Average, 18.6		9.4	177.1	70.1	68.8/63.1		106.3/96.9	40.0/49.4	0.092	0.082
P Value (Dominant vs Non-Dominant)					0.5022		0.0179	0.2333	0.2443	

ER: External rotation.

IR: Internal rotation.

R: Right.

L: Left.

T: Thoracic.

### Joint contact area

**Figure 3:**
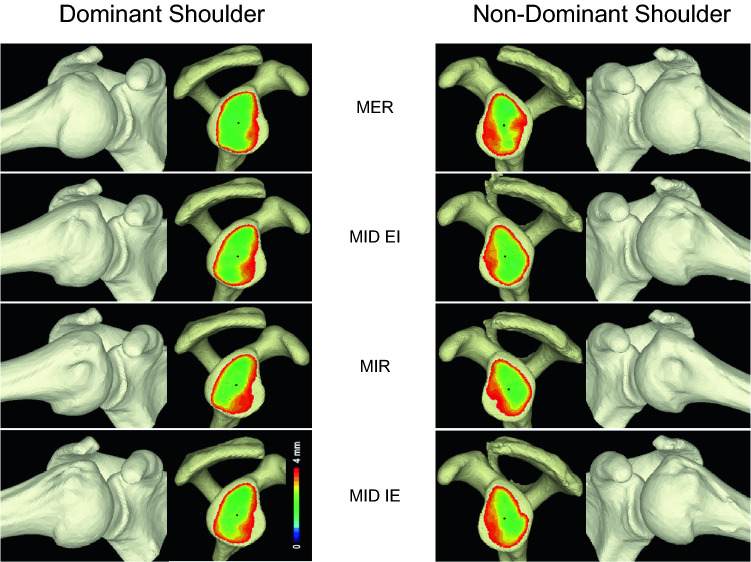
The averaged bone models and glenohumeral contact area. Black dot, center of glenohumeral contact area; MER, maximum external rotation; MIR, maximum internal rotation; MID EI, midpoint from MER to MIR; MID IE, midpoint from MIR to MER.

There was no significant difference in mean glenohumeral contact area between the dominant and non-dominant sides (Fig. 3). We normalized using body height and glenoid total area, and these data showed no significant difference in raw data (data not shown). There was no significant difference in mean glenohumeral contact area during glenohumeral joint rotation: the mean value at maximum external rotation was 692.4 ± 96.2 mm^2^ and 663.5 ± 69.2 mm^2^, and the mean value at maximum internal rotation was 663.2 ± 115.4 mm^2^ and 656.4 ± 101.3 mm^2^, for the dominant and non-dominant sides, respectively. The mean value at midpoint from maximum external rotation to maximum internal rotation was 663.7 ± 107.6 mm^2^ and 654.2 ± 89.5 mm^2^, and the mean value at midpoint from maximum internal rotation to maximum external rotation was 659.5 ± 117.4 mm^2^ and 649.7 ± 80.8 mm^2^, for the dominant and non-dominant sides, respectively. Anterior portion of the glenoid showed no contact because of the threshold.

**Figure 4:**
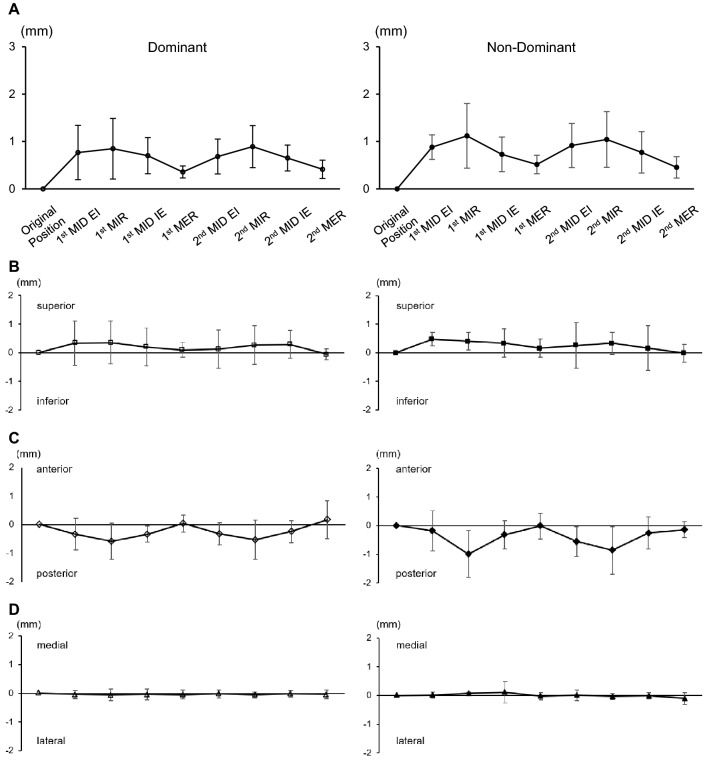
Distance of glenoid contact area centroid from original position to each position. (**A**) Absolute value of dominant shoulder and non-dominant shoulder. (**B**) Superior/inferior directions, (**C**) anterior/posterior directions, (**D**) medial/lateral directions. MER; Maximum external rotation. MIR; Maximum internal rotation. MID EI; Midpoint from MER to MIR, MID IE; Midpoint from MIR to MER.

Figure 4A shows translation of the center of glenohumeral contact area from the original position during simulated pitching motion. On both the dominant and non-dominant sides, translation of the center of glenohumeral contact area increased gradually until maximum internal rotation, and then decreased to the point of maximum external rotation. There was no significant difference between the dominant and non-dominant sides in terms of change in the translation of the center of glenohumeral contact area. On the dominant side, translation of the center of glenohumeral contact area from the original position was 0.89 ± 0.44 mm to the first maximum internal rotation. On the non-dominant side, translation of the center of glenohumeral contact area was 1.12 ± 0.68 mm from the original position to the first maximum internal rotation.

When the translation was decomposed into superior, anterior, and medial directions (Fig. 4B-D), the direction toward superior and inferior translation was stable during glenohumeral joint rotation (Fig. 4B). Translation in the medial and lateral direction was also stable (Fig. 4D). That toward anterior and posterior directions was more changeable, and gradually translated posteriorly from the original position to maximum internal rotation (Fig. 4C). The posterior translation of the center of glenohumeral contact area from the original position to the first maximum internal rotation was 0.58 ± 0.63 mm on the dominant side and 0.99 ± 0.82 mm on the non-dominant side (Fig. 4C).

### Center of humeral head

**Figure 5:**
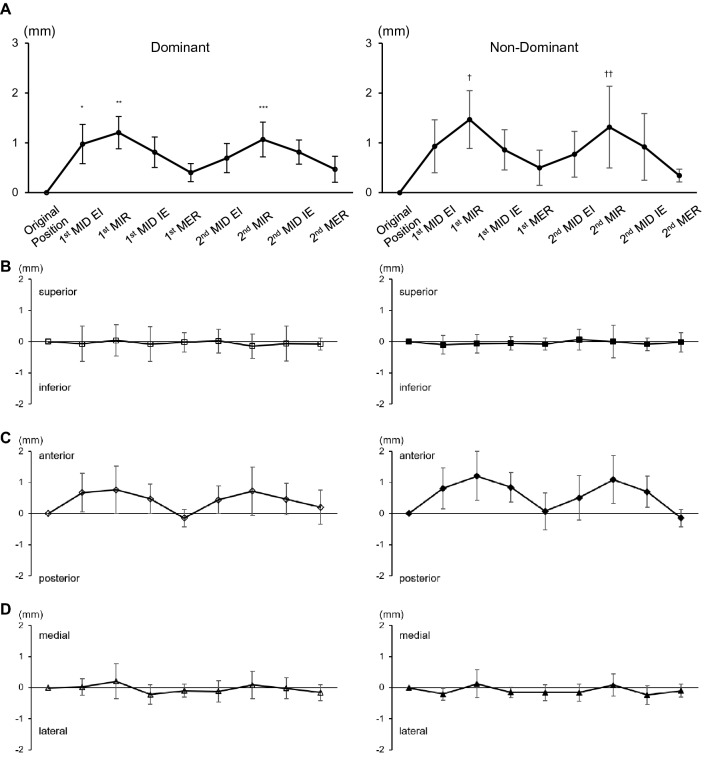
Distance of the center of humeral head from the original position to all other positions. (**A**) Absolute value of dominant shoulder and non-dominant shoulder. (**B**) Superior/inferior directions, (**C**) anterior/posterior directions, (**D**) medial/lateral directions. MER, maximum external rotation; MIR, maximum internal rotation; MID EI, midpoint from MER to MIR; MID IE, midpoint from MIR to MER. *P = 0.0141 vs 1st MER, 0.0316 vs 2nd MER, **P = 0.0001 vs 1st MER, 0.0003 vs 2nd MER, ***P = 0.0024 vs 1st MER, 0.0059 vs 2nd MER, †P = 0.0250 vs 1st MER, 0.0050 vs 2nd MER, ††P = 0.0241 vs 2nd MER.

The translations of the center of humeral head from the original position to all other positions are shown in Fig. 5A. On both the dominant and non-dominant sides, translation of the center of humeral head increased gradually until maximum internal rotation, and then decreased to the point of maximum external rotation. On the dominant side, translation of the center of humeral head from the original position was 1.20 ± 0.32 mm to the first maximum internal rotation, which was significantly higher than that to the first maximum external rotation (0.40 ± 0.18 mm, p = 0.0001) and that to the second maximum external rotation (0.45 ± 0.22 mm, *p* = 0.0003). Translation of the center of humeral head from the original position was 1.07 ± 0.35 mm to the second maximum internal rotation, which was significantly higher than that to the first maximum external rotation (p = 0.0024) and to the second maximum external rotation (*p* = 0.0059). Translation of the center of humeral head from the original position to the first midpoint of maximum external rotation to internal rotation (0.97 ± 0.39 mm) was significantly higher than that to the first maximum external rotation (*p* = 0.0141) and to the second maximum external rotation (*p* = 0.0316) (Fig. 5A). The same trend was observed on the non-dominant side, where translation of the center of humeral head from the original position was 1.47 ± 0.58 mm to the first maximum internal rotation, which was significantly higher than that to the first maximum external rotation (0.50 ± 0.35 mm, *p* = 0.0250) and that to the second maximum external rotation (0.34 ± 0.13 mm, *p* = 0.0050). Translation of the center of humeral head from the original position was 1.32 ± 0.82 mm to the second maximum internal rotation, which was significantly higher than that to the second maximum external rotation (p = 0.0241) (Fig. 5A).

When the translation was decomposed into superior, anterior, and medial directions (Fig. 5B-D), the direction toward superior and inferior translation was stable during glenohumeral joint rotation (Fig. 5B). Translation in the medial and lateral directions was also stable (Fig. 5D). That toward anterior and posterior directions was more changeable, and gradually translated anteriorly from the original position to maximum internal rotation (Fig. 5C). The anterior translation of the center of humeral head from the original position to the first maximum internal rotation was 0.76 ± 0.75 mm on the dominant side and 1.21 ± 0.78 mm on the non-dominant side (Fig. 5C).

### Correlation between the center of glenohumeral contact area and the center of humeral head

Table 2 shows the correlation between the center of glenohumeral contact area and the humeral head. A high positive correlation was found between the distance of center of glenohumeral contact area and the center of humeral head (dominant, r = 0.9763; non-dominant, r = 0.9535). There was no correlation between the superior translation of center of glenohumeral contact area and the center of humeral head (dominant, r = -0.0914; non-dominant, r = -0.3180). There was no correlation between the medial translation of the center of glenohumeral contact area and the center of humeral head (dominant, r = -0.3287; non-dominant, r = 0.0874). A high negative correlation was found between the anterior translation of the center of glenohumeral contact area and the center of humeral head (dominant, r = -0.7855; non-dominant, r = -0.7552).

**Table 2:** Correlation between the Center of Glenohumeral Contact Area and the Center of Humeral Head.

Shoulder positions	Total translation, mm				Superior translation, mm				Anterior translation, mm				Medial translation, mm			
	Dominant		Non-Dominant		Dominant		Non-Dominant		Dominant		Non-Dominant		Dominant		Non-Dominant	
	CGCA	CHH	CGCA	CHH	CGCA	CHH	CGCA	CHH	CGCA	CHH	CGCA	CHH	CGCA	CHH	CGCA	CHH
Original	0	0	0	0	0	0	0	0	0.0000	0.0000	0.0000	0.0000	0.0000	0.0000	0.0000	0.0000
1^st^ MID EI	0.7681	0.9746	0.7681	0.9746	0.3374	− 0.0726	0.4764	− 0.1068	− 0.3343	0.7964	− 0.1791	0.8104	− 0.0424	0.0184	0.0148	− 0.2040
1^st^ MIR	0.8494	1.2049	0.8494	1.2049	0.3575	0.0418	0.3969	− 0.0675	− 0.5796	0.6058	− 0.9913	1.2999	− 0.0594	0.2014	0.0756	0.1257
1^st^ MID IE	0.7011	0.8119	0.7011	0.8119	0.2022	− 0.0790	0.3314	− 0.0468	− 0.3382	0.4472	− 0.3172	0.7955	− 0.0395	− 0.2160	0.1159	− 0.1509
1^st^ MER	0.3578	0.4022	0.3578	0.4022	0.0989	−0.0199	0.1614	− 0.0814	0.0330	− 0.2294	− 0.0107	0.1609	− 0.0426	− 0.0980	− 0.0313	− 0.1570
2^nd^ MID EI	0.6831	0.6933	0.6831	0.6933	0.1234	0.0247	0.2558	0.0692	− 0.3244	0.4533	− 0.5495	0.4900	− 0.0198	− 0.1178	0.0126	− 0.1585
2^nd^ MIR	0.8921	1.0669	0.8921	1.0669	0.2684	− 0.1472	0.3310	− 0.0008	− 0.5386	0.5449	− 0.8590	0.9150	− 0.0539	0.0881	− 0.0328	0.0832
2^nd^ MID IE	0.6516	0.8146	0.6516	0.8146	0.2910	− 0.0639	0.1648	− 0.0803	− 0.2440	0.5116	− 0.2497	0.8015	− 0.0176	− 0.0168	− 0.0122	− 0.2345
2^nd^ MER	0.4138	0.4480	0.4138	0.4480	− 0.0517	− 0.0814	− 0.0199	− 0.0199	0.1674	0.1674	− 0.1401	− 0.1955	− 0.0390	− 0.1570	− 0.0980	− 0.0980
Correlation	0.9763		0.9535		− 0.0914		− 0.3180		− 0.7855		− 0.7552		− 0.3287		0.0874	

CGCA: Center of glenohumeral contact area.

CHH: Center of humeral head.

MER: Maximum external rotation.

MIR: Maximum internal rotation.

MID EI: Midpoint from MER to MIR.

MID IE: Midpoint from MIR to MER.

### Correlation between translation the center of humeral head and humeral head oblateness

Table 3 shows the correlation between translation of the center of humeral head and humeral head oblateness. A high positive correlation was found between the translation of the center of humeral head and humeral head oblateness (dominant, r = 0.6530; non-dominant, r = 0.6885).

**Table 3:** Correlation between Translation the Center of Humeral Head and Humeral Head Oblateness.

Case		Case 1	Case 2	Case 3	Case 4	Case 5	Case 6	Case 7	Case 8	Correlation
Dominant	Translation of the CHH from original to MIR, mm	0.5811	1.4523	0.9908	1.4776	1.6252	1.0895	1.4041	1.0183	0.6530
	Humeral head oblateness	0.0778	0.0906	0.0911	0.1221	0.1132	0.0615	0.0904	0.0873	
Non-Dominant	Translation of the CHH from original to MIR, mm	1.4651	1.3032	2.2808	2.2978	0.6640	1.0895	1.8157	0.8302	0.6885
	Humeral head oblateness	0.0764	0.0743	0.0896	0.1104	0.0667	0.0866	0.0722	0.0773	

CHH: Center of humeral head.

MIR: Maximum internal rotation.

## Discussion

To the best of our knowledge, this is the first study to evaluate 4D alteration in glenohumeral contact area and translation of the center of humeral head during active cocking motion in vivo. The present findings demonstrated that the glenohumeral contact area altered during active cocking motion. In addition, the center of humeral head translated anteriorly during active internal rotation and posteriorly during active external rotation in abduction on both of the dominant and non-dominant sides.

Recent studies have demonstrated that glenohumeral contact area varies depending on the shoulder position. In a study of cadaveric shoulders, Greis^29^ et al. reported that glenohumeral contact area increased 5.0% from 30° of abduction and neutral position to 90° abduction and neutral position. Bhatia^30^ et al. reported that glenohumeral contact area decreased from 5.44 cm^2^ in 60° of abduction and neutral position to 4.05 cm^2^ in 60° abduction and 90° external rotation. In a study of in vivo shoulders, previous study showed that glenohumeral contact area changed with and without muscle contraction using MRI static position^17^. However, most of these measurements were obtained in a series of static poses. Because internal forces differ between static and dynamic tasks, and the muscular pattern affects the glenohumeral contact area, we developed a protocol based on 4D CT and a customized computer program to measure 3D glenohumeral kinematics in dynamic motion. Recent developments have enabled multidetector CT scanners with wide CT gantries to obtain multiple scans in 0.1 s, and thus 4D CT analysis of joint motion^31,32^. We used this technology to evaluate the glenohumeral contact area quantitatively during active cocking motion with the shoulder abducted in baseball pitchers. The glenohumeral contact area in these intact shoulders tended to decreased during active abduction–internal rotation, but the change in glenohumeral contact area during dynamic motion was not statistically significant. The present results might be affected by the internal forces.

Excessive anterior translation of the humeral head during the throwing motion is thought to increase the risk of injury^33,34^. Glenohumeral translations has been a controversial topic as the range in reported values has been only a few millimeters below the limits of accuracy^35^. Furthermore, a previous study has reported that the center of humeral head translated 3.4 mm posteriorly during glenohumeral external rotation with the shoulder in the abduction^21^. The results of the present study found that the center of humeral head translated 1.20 ± 0.32 mm anteriorly during the cocking motion. Regarding the direction of humeral head translation, the current results are comparable to those of previous reports and our value of SD were smaller than that of previous reports. To better understand the thrower’s shoulder pathologies, further studies should analyze more data obtained from players with pathological conditions.

**Figure 6:**
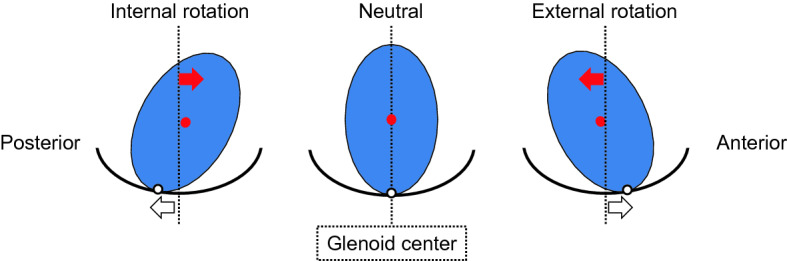
Influence of oblateness of humeral head on translation of the center of glenohumeral contact area (CGHCA; white dot) and the center of humeral head (CHH; red dot) during shoulder rotation in axial plane. At internal rotation position, CGHCA translated posterior (white arrow) whereas CHH translated anterior (red arrow). At external rotation position, CGHCA translated anterior (white arrow) whereas CHH translated posterior (red arrow).

The current study showed that the center of the glenohumeral joint contact area translated posteriorly during shoulder internal rotation, whereas the center of humeral head translated anteriorly. This diametric translation appears to be caused by oblateness of the humeral head (Fig. 6). The current study showed a positive correlation between oblateness and translation of the center of humeral head. This result indicates diametric translation of the center of glenohumeral contact area and the center of humeral head. The oblateness of the humeral head may cause this diametric translation.

There are some limitations of the present study. First, we did not scan the entire scapula and humerus. Second, we used a surface registration technique. Third, since all participants were healthy pitchers, there may not have been a significant difference between the dominant and non-dominant sides. Although there was no significant difference in the mean first ER or second IR between the dominant and non-dominant sides, the values were comparable to those in past studies. Finally, our analysis was not based on direct measurement of pitching motion; however, the shoulders consistently showed a characteristic pattern of glenohumeral kinematic changes, and the present results appear to accurately represent the glenohumeral kinematics of normal shoulders in the cocking motion.

In conclusion, 4D CT scanning and the tracer software for bone surface modeling of the glenohumeral joint show promise for evaluation of glenohumeral micromotion.
